# Role of exosomal microRNAs in lung cancer biology and clinical applications

**DOI:** 10.1111/cpr.12828

**Published:** 2020-05-11

**Authors:** Chengping Hu, Silke Meiners, Christina Lukas, Georgios T. Stathopoulos, Jie Chen

**Affiliations:** ^1^ Department of Respiratory Medicine Xiangya Hospital Central South University Changsha China; ^2^ Comprehensive Pneumology Center and Institute for Lung Biology and Disease University Hospital Ludwig‐Maximilians University of Munich Munich Germany; ^3^ Helmholtz Center Munich Munich Germany; ^4^ Laboratory for Molecular Respiratory Carcinogenesis Department of Physiology Faculty of Medicine Biomedical Sciences Research Center University of Patras Rio Greece

**Keywords:** biomarker, exosomal miRNAs, exosomes, lung cancer, therapy

## Abstract

Exosomes, small extracellular vesicles ranging from 30 to 150 nm, are secreted by various cell types, including tumour cells. Recently, microRNAs (miRNAs) were identified to be encapsulated and hence protected from degradation within exosomes. These exosomal miRNAs can be horizontally transferred to target cells, in which they subsequently modulate biological processes. Increasing evidence indicates that exosomal miRNAs play a critical role in modifying the microenvironment of lung cancers, possibly facilitating progression, invasion, angiogenesis, metastasis and drug resistance. In this review, we summarize the novel findings on exosomal miRNA functions during lung cancer initiation and progression. In addition, we highlight their potential role and challenges as biomarkers in lung cancer diagnosis, prognosis and drug resistance and as therapeutic agents.

## 1 INTRODUCTION

Lung cancer is the second most common and the most lethal cancer worldwide, accounting for 11.6% of all new cancer cases and 19.8% of all cancer‐related deaths.^1^ Recent research has fostered new insights into lung cancer biology, and considerable progress has been made in the field of novel biomarker‐targeted therapies, including molecular therapies targeting epidermal growth factor receptor (EGFR), anaplastic lymphoma kinase (ALK) and proto‐oncogene B‐Raf (BRAF), ^2^, ^3^ and immunotherapies with checkpoint inhibitors targeting the programmed cell death 1 (PD‐1) and programmed death‐ligand 1 (PD‐L1) pathways.^4^ However, lung cancer still has one of the lowest 5‐year survival rates (only 18%) of all cancer types.^5^ The high mortality is attributed to the fact that most lung cancers are diagnosed at advanced stages with limited treatment options and 5‐year survival rates of only 4%.^5^ Therefore, the identification of reliable diagnostic biomarkers and effective therapeutic strategies is an unmet medical need in lung cancer. Exosomes, small extracellular vesicles ranging between 30 and 150 nm in size, are secreted by various cell types, including tumour cells.^6^ It has been shown that they deliver their cargo (proteins or nucleic acids) to specific cell types, which subsequently act as important messengers in cancer.^7^ It was also reported that cancer cells produce 10‐fold more exosomes than normal cells and that exosomes derived from cancer cells can facilitate cellular communication via delivery of growth factors, chemokines, miRNAs, etc.^8^, ^9^ Interestingly, researchers have shown that exosomes derived from different cells possess unique mRNA and miRNA expression profiles that may differ from their donor cells.^10^ Furthermore, accumulating evidence reveals that cancer‐derived exosomal miRNAs play important roles in the recruitment and reprogramming of constituents of the tumour environment.^11^ Therefore, exosomal miRNAs are regarded as potentially ideal non‐invasive tools for early diagnosis as well as therapeutic targets, since they contain key information on signalling pathways related to tumour biological responses. In this review, we summarize the recent findings on exosomal miRNA involvement in cancer initiation and progression, mainly focusing on lung cancer. Furthermore, we highlight the potential future use of exosomal miRNAs as biomarkers and therapeutic targets or agents in lung cancer.

## 2 PROPOSED ROLE OF EXOSOMAL MIRNAS IN LUNG CANCER

**FIGURE 1:**
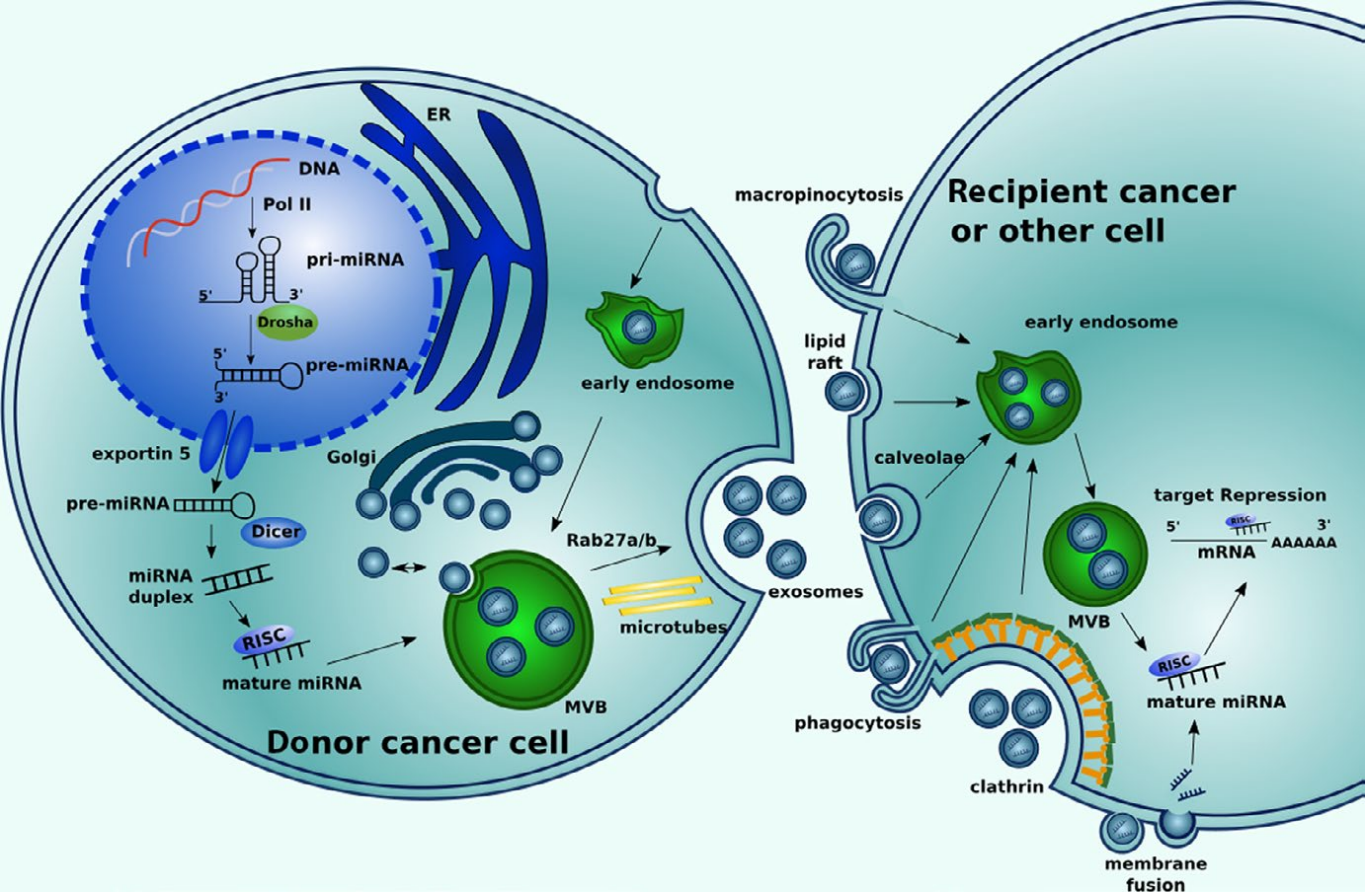
Biology of exosomal miRNAs. In animals, microRNA (miRNA) genes are transcribed into primary miRNAs (pri‐miRNAs) by RNA polymerase II (Pol‐II) and then processed by the Drosha complex to form precursor miRNAs (pre‐miRNAs), which are exported into the cytoplasm by the exportin5 complex. The pre‐miRNAs are digested by the Dicer complex to become double‐stranded miRNAs. With the help of a helicase, they are then turned into single‐stranded mature miRNAs. Mature miRNAs are sorted into multivesicular bodies (MVBs). The MVBs are then transported along microtubules to the plasma membrane and released as exosomes. Exosomes with special miRNAs from the parent cell can interact with the recipient cell through different ways, such as by fusion via clathrin‐dependent endocytosis, clathrin‐independent endocytosis (micropinocytosis or phagocytosis), caveolae‐mediated endocytosis or lipid raft‐dependent endocytosis. Once exosomes enter recipient cells, exosomal miRNAs may act in target repression

Exosomes are membrane‐encapsulated vesicles present in many biological fluids. They are important mediators of cell‐to‐cell communication and regulators of biological processes. Exosomes contain multifaceted cargoes, including proteins, lipids, DNAs, mRNAs and miRNAs.^12^ Among these molecules, miRNAs, which are short and endogenous non‐coding RNAs, are the most intriguing and extensively studied due to their powerful regulatory role in gene expression at the post‐transcriptional and translational levels^12^, ^13^ (Figure 1). In the tumour microenvironment, including that of lung cancer, transfer of exosomal miRNA between cancer and stromal cells has been demonstrated to be linked with cancer initiation and progression.^14^, ^15^, ^16^, ^17^, ^18^

**FIGURE 2:**
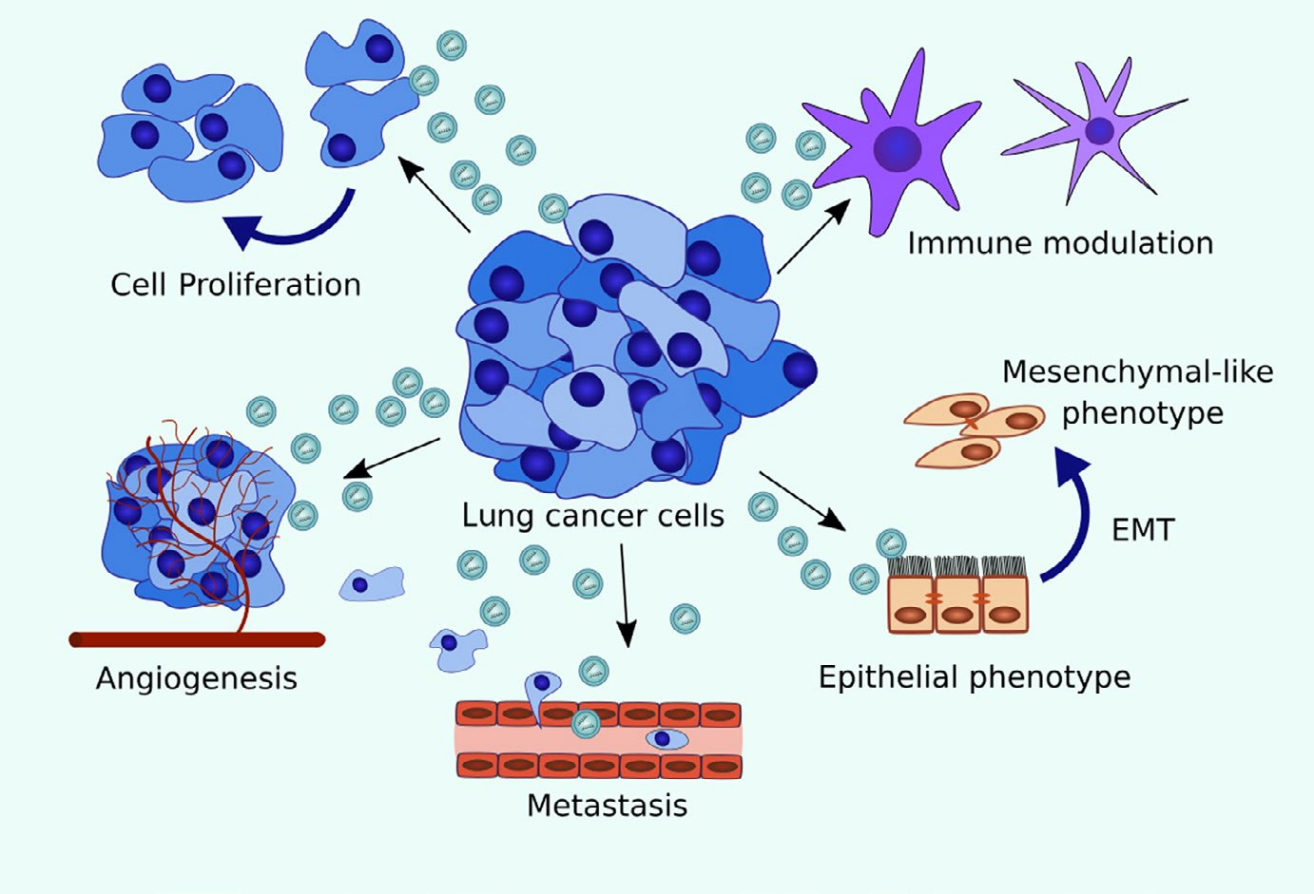
Exosomal miRNAs in lung cancer. Lung cancer cells export exosomal miRNAs to parent cells to affect their proliferation, angiogenesis, EMT and metastasis. Lung cancer cells are also able to export exosomal miRNAs to immune cells and influence the function of immune cells

In patients with lung cancer, the concentrations of both circulating exosomes and exosomal miRNA are elevated compared with the respective concentrations in controls.^19^ Moreover, exosomal miRNA levels are elevated in both plasma and bronchoalveolar lavage (BAL) samples from patients with non–small‐cell lung cancer (NSCLC) compared with those in non‐tumour patients.^20^ In addition, recent functional studies identified relationships between exosomal miRNAs and lung cancer hallmark pathways ranging from metabolism to intercellular communication (Figure 2). To this end, exosomal miRNAs have been implicated in a series of biological processes in lung cancer, including proliferation, angiogenesis and metastasis (Table 1). Moreover, exosomal miRNAs have been found to affect the lung tumour microenvironment and to signal to the immune system. As detailed below, the exosomal miRNA effects in lung cancer are largely similar to those of miRNAs of non‐exosomal origin.

**TABLE 1:** Studies on exosomal miRNAs in basic research of lung cancer

Exosomal miRNAs	Donor	Recipient	Target(s)	Function	Processes involved in	Reference
miR‐512	A549 cells	A549 cells	TEAD4	Reduce cell proliferation	Proliferation	[16]
miR‐208a	A549 cells	A549 cells	p21	Activation of the AKT/mTOR pathway	Proliferation	[21]
miR‐96	H1299 cells	A549 cells	LMO7	‐	Proliferation	[22]
miR‐9	H1299 cells	Endothelial cells	SOCS5	Activation of JAK‐STAT pathway.	Angiogenesis	[24]
miR‐210	A549 cells	Human umbilical vein endothelial cell (HUVECs)	EphA3	‐	Angiogenesis	[25]
miR‐21	Cigarette smoke extract (CSE)‐transformed human bronchial epithelial (HBE) cells	Human umbilical vein endothelial cells	VEGF	Increase VEGF expression and induce angiogenesis	Angiogenesis	[17]
miR‐23a	Human lung cancer cells	Human umbilical vein endothelial cells	Prolyl hydroxylase 1 and 2, ZO‐1	‐	Angiogenesis	[26]
miR‐23a	E‐phenotype A549 cells	M‐phenotype A549 cells	‐	‐	EMT	[31]
miR‐193a‐3p, miR‐210‐3p and miR‐5100	Hypoxic bone marrow‐derived mesenchymal stem cells	NSCLC cell lines including H358, A549, H460	STAT3 signalling	Promote invasion of lung cancer cells	EMT	[15]
miR‐494 and miR‐542‐3p	Adenocarcinoma cells	Lymph node stromal cells and lung fibroblasts	Cadherin‐17	Affect proteases, adhesion molecules, chemokine ligands, cell cycle– and angiogenesis‐promoting genes, and genes engaged in oxidative stress response	Metastasis	[35]
miR‐21	A549 cells	Bone marrow monocyte	Pdcd4	Promote effects on osteoclastogenesis	Metastasis	[18]
miR‐21 and miR‐29a	A549 and SK‐MES cells	Human PBMCs or murine peritoneal macrophage	TLR7 and TLR8	Trigger a TLR‐mediated prometastatic inflammatory response	Metastasis	[36]
miR‐192	A549, mock M1 and miR‐192 M1 overexpressing cells.	Human umbilical vein endothelial cells	‐	Abrogation of the angiogenic programme by repression of proangiogenic IL‐8, ICAM and CXCL1.	Metastasis	[37]
miR‐193a‐3p, miR‐210‐3p and miR‐5100	Hypoxic bone marrow‐derived mesenchymal stem cells	NSCLC cell lines including H358, A549, H460	STAT3 signalling	Promote invasion of lung cancer cells	Metastasis	[15]
miR‐100‐5p	Cisplatin‐resistant lung cancer cells A549	Lung cancer cells A549	mTOR	Modulate sensitivity to DDP	Drug resistance	[39]
miR‑222‐3p	Gemcitabine‐resistant A549	Lung cancer cells A549	SOCS3	Promote migration, invasion, anoikis resistance and gemcitabine resistance	Drug resistance	[40]
miR‑96	H1299 cells	A549 cells	LIM‐domain only protein 7	Alter the chemotherapeutic sensitivity of lung cancer cells	Drug resistance	[22]
miR‐146a‐5p	Cisplatin‐resistant lung cancer cells A549	Lung cancer cells A549	Autophagy‐related protein 2 (Atg2)	Increasing chemosensitivity of NSCLC to cisplatin by inhibiting autophagy	Drug resistance	[41]
miR‐512 and miR‐373	5′aza‐deoxycytidine plus trichostatin A‐treated A549	Lung cancer cells A549	miR‐512 targets TEAD4; miR‐373 targets RelA and PIK3CA	Sensitizes lung cancer cells to cisplatin and restricts tumour growth	Drug resistance	[16]
miR‐21	Gefitinib‐resistant H827R cells	Gefitinib‐sensitive HCC827 cells	‐	Activate the AKT signalling	Drug resistance	[42]
miR‐214	Gefitinib‐resistant PC9GR cells	PC9 cells	‐	Acquisition of gefitinib resistance	Drug resistance	[43]

### 2.1 Exosomal miRNAs and cellular proliferation in lung cancer

Proliferation, characterized by altered expression and/or activity of cell cycle‐related proteins, plays a critical role in cancer development and progression.^14^ Exosomes transfer genetic information between cells in the tumour environment via exosomal miRNAs, thereby promoting lung cancer cell proliferation. For example, Harel et al found that exosomal miR‐512 halted lung tumour cell proliferation by targeting TEA domain family member 4 (TEAD4), indicating that miR‐512 possesses tumour‐suppressive effects.^16^ Moreover, miR‐208a packaged in exosomes from A549 NSCLC cells was demonstrated to act as a transfer messenger and to target p21 with corresponding activation of the AKT/mechanistic target of rapamycin (mTOR) pathway, thereby inhibiting NSCLC cell proliferation.^21^ In addition, miR‐96–containing exosomes from H1299 cells could promote cell proliferation by directly targeting and inhibiting LIM‐domain only protein 7 (LMO7) expression, demonstrating their tumour‐promoting role.^22^

### 2.2 Exosomal miRNAs and angiogenesis in lung cancer

Tumour angiogenesis, essential for tumour growth and metastasis, is modulated by exosomes released by different cell types that act as cell‐to‐cell mediators.^23^ To this end, one study indicated that exosomal miR‐9 stimulates angiogenesis by activating the JAK/STAT signalling pathway.^24^ Tissue inhibitor of metalloproteinases‐1 (TIMP‐1) upregulated exosomal miR‐210 derived from lung adenocarcinoma (LUAD) samples and ultimately stimulated angiogenesis in stromal cells.^25^ In addition, Liu et al found that exosomal miR‐21 led to activation of signal transducer and activator of transcription (STAT) 3, increasing VEGF expression and inducing angiogenesis and malignant transformation of human bronchial epithelial cells (HBECs).^17^ Finally, exosomal miR‐23a derived from lung cancer cells was demonstrated to enhance tumour angiogenesis under both normoxic and hypoxic conditions, indicating that lung cancer cells transmit genetic material to distant endothelial cells.^26^

### 2.3 Exosomal miRNAs in EMT and metastasis in lung cancer

Metastasis is a complex process that requires cancer cells to invade blood or lymph vessels, disseminate to a new location and establish colonies at the new site.^27^ Epithelial‐to‐mesenchymal transition (EMT), characterized by epithelial cells losing cell‐to‐cell adhesion and cellular polarity and acquiring a mesenchymal migratory and invasive phenotype,^28^ is essential for tumour progression and metastasis.^29^, ^30^ Several lines of evidence have confirmed that exosomes and their cargo (especially miRNAs) play critical roles in different steps of the metastatic process. For example, it was demonstrated that exosomal miR‐23a was significantly increased after induction of EMT with transforming growth factor (TGF)‐β1 in A549 cells.^31^ Moreover, Tang et al reported changes in the exosomal miRNA profile upon EMT in the human NSCLC cell lines A549 and H1299 and that some miRNAs specifically contained in exosomes derived from mesenchymal phenotype cells were associated with EMT and metastasis.^32^ However, the mechanisms by which miRNAs transferred by exosomes affect tumour metastasis remain poorly understood.^33^ There appear to be three mechanisms of exosomal miRNA transfer during lung cancer metastasis.^34^ First, exosomes from invasive cells transfer miRNAs to less invasive cells, thereby altering the status of recipient cells and ultimately prompting metastasis. For example, exosomes derived from adenocarcinoma cells were able to target non‐transformed cells (lymph node stromal cells and lung fibroblasts) and to modulate pre‐metastatic organ cells predominantly through transferred miR‐494 and miR‐542‐3p.^35^ Another example is LUAD cell‐derived exosomal miR‐21, which was shown to be transferred to osteoclast progenitor cells and to target programmed cell death 4 (PDCD4) to facilitate osteoclastogenesis.^18^ Second, primary tumour cells can contact other cells in the tumour microenvironment through exosomal miRNA. For example, lung cancer cells activate Toll‐like receptors TLR7 and TLR8 on immune cells via exosomal miR‐21 and miR‐29a, leading to tumour growth and metastasis.^36^ Additionally, lung cancer cells also target endothelial cells via exosomal miR‐192 to abrogate their angiogenic programme and influence bone metastatic colonization.^37^ Third, normal cells release exosomes with special miRNAs to change the behaviour of tumour cells. For example, exosomes derived from hypoxic bone mesenchymal stem cells (BMSCs) transferred miR‐193a‐3p, miR‐210‐3p and miR‐5100 to lung cancer cells and activated STAT3 signalling‐induced EMT, promoting metastasis.^15^

### 2.4 Exosomal miRNAs in drug resistance in lung cancer

The development of drug resistance is one of the main factors of poor prognosis in lung cancer. Lung cancer patients easily develop resistance to not only conventional chemotherapy drugs, but also molecular targeted drugs, such as epidermal growth factor receptor tyrosine kinase inhibitors (EGFR‐TKIs). Therefore, exploring the potential mechanisms that impair drug efficacy will help to improve cancer treatments.

Exosomes mediate intercellular communication by transferring proteins and nucleic acids to target cells. Recent evidence has revealed that exosomes involved in mediating drug resistance via horizontal transfer of specific bioactive cargoes, especially miRNAs. Qin et al demonstrated that 11 upregulated miRNAs and 31 downregulated miRNAs were significantly different between A549/cisplatin (DDP) cells and A549 cells, as well as their respective exosomes by an miRNA‐chip approach.^38^, ^39^ Through target gene prediction and pathway analysis, most of the miRNAs were found to be linked to drug resistance. In vitro and in vivo A549 xenograft studies demonstrated that exosomes could transfer miR‐100‐5p to recipient cells, with mTOR as its potential target gene, and thereby modulate cell sensitivity to DDP.^39^ Wei et al also revealed that gemcitabine‐resistant A549 (A549‐GR) cells could effectively assemble miR‐222‐3p into A549‐GR–secreted exosomes, which could then be transported into parental sensitive cells and promote migration, invasion, anoikis resistance and gemcitabine resistance by directly targeting the promoter of SOCS3.^40^ Furthermore, the expression of miR‐96 was significantly higher in lung cancer than in normal lung tissue. The same tendency was observed in exosomes isolated from lung cancer patients and controls. MiR‐96 could alter the chemotherapeutic sensitivity of lung cancer cells by downregulating the drug resistance‐related gene LMO7.^22^ Additionally, the levels of exosomal miR‐146a‐5p were also found to be significantly lower in advanced NSCLC patients with higher recurrence rates than in those with lower recurrence rates. Exosomal miR‐146a‐5p was demonstrated to be linked with DDP responses by targeting autophagy‐related protein 2 (Atg2) to inhibit autophagy.^41^ Finally, exosome‐derived miR‐512 and miR‐373 were also shown to be associated with increased sensitivity to DDP and suppression of tumour progression.^16^

In addition, exosomal miRNAs were also involved in resistance to EGFR‐TKIs. For example, Jing et al^42^ showed that exosomal miR‐21 could be transferred from gefitinib‐resistant H827R cells to gefitinib‐sensitive HCC827 cells, where it subsequently activated AKT signalling and lead to gefitinib resistance. Our group also reported that the levels of miR‐214 were significantly higher in gefitinib‐resistant PC9GR cells and their derived exosomes than in gefitinib‐sensitive PC9 cells and their derived exosomes, respectively. When exosomal miR‐214 was transferred from PC9GR cells to PC9 cells, gefitinib resistance was acquired in PC9 cells. However, when PC9GR cell‐derived exosomes were transfected with an miR‐214 antagomir, the acquisition of gefitinib resistance was reversed.^43^

### 2.5 Exosomal miRNAs and immunity in lung cancer

Immune checkpoint molecules play a critical role in regulating the immune system to maintain self‐tolerance and prevent autoimmunity.^44^ Targeting immune checkpoint molecules principally represented by programmed cell death protein 1 (PD‐1) and its ligand PD‐L1 has resulted in improved survival for advanced NSCLC patients.^45^ A network of miRNAs has been demonstrated to control immune checkpoint‐related processes. For example, miR‐34 was shown to be controlled by p53 and directly binds to the PD‐L1 3'‐UTR and represses its expression in NSCLC models.^46^ MiR‐200 was also revealed to control PD‐L1 expression.^47^

Increasing evidence indicates that exosomes participate in tumour progression by delivering immunosuppressive molecules and factors.^48^ Exosomal miRNAs are important carriers that can influence the function of immune cells, including dendritic cells (DCs) and T‐lymphocytes, in cancer.^14^ For example, as discussed above, exosomes derived from lung cancer cells transferred miR‐21/29a to activate TLR7 and TLR8 on immune cells, which may contribute to tumour growth and metastasis.^36^ Additionally, Yin et al showed that human cancer cells, including lung cancer cells, delivered miR‐214 to recipient CD4^+^ T cells via exosomes, which ultimately decreased phosphatase and tensin homolog (PTEN) expression and promoted regulatory T cell (Treg) expansion and tumour growth.^49^

### 2.6 Exosomal miRNA from non‐tumour cells in lung cancer

Although numerous studies have focused on the role of only exosomal miRNAs derived from cancer cells, an increasing number of studies have focused on those exosomal miRNAs derived from non‐cancer cells within the tumour microenvironment. After being transferred from non‐cancer cells to recipient cells, exosomal miRNAs can effectively influence the recipient cell phenotype (including epithelial and stromal cells) and then play a crucial role in the growth and progression of lung malignancies by modulating a wide range of pathways. For instance, by secreting cigarette smoking‐induced exosomes containing miR‐21, human bronchial epithelial cells (HBECs) have been shown to enhance VEGF levels through STAT3 deregulation, thereby promoting angiogenesis and tumour growth.^17^ Similarly, Fujita and colleagues also reported that the transfer of miR‐210 in cigarette smoke extract (CSE)‐induced HBEC‐derived exosomes promoted the myofibroblast differentiation and autophagy of lung fibroblasts (LFs).^50^ Moreover, exosome transfer of miR‐223 from platelets to lung cancer cells has been reported to modulate invasion through the suppression of EPB41L3.^51^ In addition, exosomal miRNAs from MSCs are also reported to be involved in tumour growth. As stated above, exosomes derived from hypoxic BMSCs were demonstrated to transfer miR‐193a‐3p, miR‐210‐3p and miR‐5100 to lung cancer cells, thus activating STAT3 signalling‐induced EMT and promoting metastasis.^15^

## 3 CLINICAL IMPLICATIONS OF EXOSOMAL MIRNAS IN LUNG CANCER

A number of studies have shown that exosomal cargoes can potentially be used as diagnostic, prognostic and predictive biomarkers for lung cancer. To date, the most widely studied exosomal cargo related to lung cancer is miRNA (Table 2).

**TABLE 2:** Studies on exosomal miRNAs as diagnostic, predictive and prognostic biomarkers in lung cancer

Exosomal miRNAs	Source	Selection method for exosomes	Selection method for Exosomal miRNA	Selection cohort	Validation cohort	Clinical value	References
miR‐205, miR‐19a, miR‐19b, miR‐30b and miR‐20a	Plasma	ExoQuick	qRT‐PCR	18 patients and 6 controls	‐	Diagnostic biomarkers of lung squamous cell carcinoma (SCC)	[52]
miR‐629, miR‐30a‐3p, miR‐100, miR‐200b‐5p, miR‐154‐3p and miR‐151a‐5p	Plasma	ExoQuick	qRT‐PCR	50 adenocarcinoma patients and 30 lung granuloma patients	‐	Diagnostic biomarkers for dividing adenocarcinoma and granuloma	[53]
miR‐19‐3p, miR‐21‐5p and miR‐221‐3p	Plasma	ExoQuick	qRT‐PCR	30 lung adenocarcinoma patients and 10 controls	Training: 42 lung adenocarcinoma patients and 32 controls Testing: 66 lung adenocarcinoma patients and 62 controls.	Diagnostic biomarkers for lung adenocarcinoma	[54]
miR‐96	Plasma	ExoQuick	qRT‐PCR	56 lung cancer patients and 19 controls	‐	Diagnostic biomarker for lung cancer	[22]
miR‐126	Plasma	Ultracentrifugation	qRT‐PCR	45 NSCLC patients and 31 controls	‐	Diagnostic biomarker for NSCLC	[55]
miR‐23a	Serum	Exosome isolation reagents (Life Technologies)	qRT‐PCR	15 lung cancer patients and 15 controls	‐	Diagnostic biomarker for lung cancer	[26]
miR‐30b/30c	Plasma	Density‐gradient ultracentrifugation	qRT‐PCR	Lung adenocarcinoma patients	‐	‐	[56]
miR‐let‐7b‐5p, miR‐let‐7e‐5p, miR‐23a‐3p and miR‐486‐5p	Plasma	Ultracentrifugation	NGS	16 lung adenocarcinoma patients in stage I, 10 SCC patients in stage I and 12 controls	10 lung adenocarcinoma patients in stage I, 10 SCC patients in stage I and 30 controls	Diagnostic biomarker for stage I NSCLC	[57]
miR‐181‐5p, miR‐30a‐3p, miR‐30e‐3p and miR‐361‐5p	Plasma	Ultracentrifugation	NGS	16 lung adenocarcinoma patients in stage I, 10 SCC patients in stage I and 12 controls	10 lung adenocarcinoma patients in stage I, 10 SCC patients in stage I and 30 controls	Diagnostic biomarkers for distinguishing lung adenocarcinoma from stage I NSCLC	[57]
miR‐10b‐5p, miR‐15b‐5p and miR‐320b	Plasma	Ultracentrifugation	NGS	16 lung adenocarcinoma patients in stage I, 12 SCC patients in stage I and 10 controls	10 lung adenocarcinoma patients in stage I, 10 SCC patients in stage I and 30 controls	Diagnostic biomarkers for distinguishing SCC from stage I NSCLC	[57]
miR‐205‐5p, miR‐483‐5p, miR‐375, miR‐200c‐3p, miR‐429, miR‐200b‐3p, miR‐200a‐3p, miR‐203a‐3p and miR‐141‐3p	Pleural effusion	Differential centrifugation	NGS	8 APE patients and 14 controls	‐	Diagnostic biomarkers for distinguishing lung adenocarcinoma over tuberculosis and other benign lesions	[58]
miR‐205‐5p and miR‐200b	Pleural effusion	Differential centrifugation	NGS	9 lung cancer patients, 9 lung pneumonia patients and 9 pulmonary tuberculosis	‐	Diagnostic biomarker for lung cancer	[59]
miR‐182 and miR‐210	Pleural effusion	Exosome isolation reagents (Invitrogen)	qRT‐PCR	41 lung adenocarcinoma patients and 15 controls	‐	Diagnostic biomarker for malignant pleural effusion (lung adenocarcinoma) over benign (non‐neoplastic) pleural effusion	[60]
miR‐200	Pleural effusion	ExoRNeasy serum plasma kit (Qiagen),	qRT‐PCR	18 lung adenocarcinoma patients and 18 controls	‐	Diagnostic biomarker for malignant pleural effusion (lung adenocarcinoma) over benign (non‐neoplastic) pleural effusion	[61]
miR‐221‐3p and miR‐222‐3p	Plasma	Exosome isolation commercial kit	qRT‐PCR	12 NCLC patients and 6 controls	‐	Predictive biomarker for osimertinib response	[64]
miR‐29a‐3p and miR‐150‐5p	Plasma	solvent‐exchange exosome isolation kit (Exiqon)	qRT‐PCR	5 NSCLC patients	‐	predictive biomarker for unexpected responses to radiation therapy	[65]
miR‐208a	Serum	Differential centrifugation	miRNA microarray	‐	‐	Predictive biomarker for radiation responses.	[21]
miR‐146a‐5p	Serum	ExoQuick exosome precipitation kit	qRT‐PCR	100 advanced NSCLC patients	‐	predictive biomarker for the efficacy of cisplatin for NSCLC patients	[41]
miR‐222‐3p	Serum	Ultracentrifugation	qRT‐PCR	50 NSCLC patients	‐	Predictive biomarker for gemcitabine sensitivity	[40]
miR‐21 and miR‐4257	Plasma	Ultracentrifugation	Chip	6 NSCLC patients	195 NSCLC patients who underwent curative surgery and 30 controls	Prognostic biomarkers for disease‐free survival (recurrence)	[69]
miR‐146a‐5p	Serum	ExoQuick exosome precipitation kit	qRT‐PCR	100 advanced NSCLC patients	‐	prognostic biomarker for recurrence	[41]
miR‐23b‐3p, miR‐10b‐5p and miR‐21‐5p	Plasma	ExoQuick Exosome Precipitation Solution kit	qRT‐PCR	10 lung adenocarcinoma and 10 controls	196 NSCLC patients	Prognostic biomarkers for overall survival	[70]
let‐7a‐5p	Serum	Ultracentrifugation	NGS	54 pneumoconiosis patients and 100 controls.	‐	Prognostic biomarkers for poor survival	[71]

### 3.1 Exosomal miRNAs as diagnostic biomarkers in lung cancer

Early diagnosis of lung cancer is critical for successful treatment.^5^ Multiple human studies have shown that exosomal miRNAs have the potential to serve as tools for the early diagnosis of lung cancer. For example, as early as 2009, Rabinowits et al demonstrated that exosomal miRNAs in NSCLC patients were similar to the miRNAs in NSCLC tissue, indicating that exosomal miRNAs could be used as markers in liquid biopsy material in NSCLC.^19^ Moreover, five different exosomal miRNAs (miR‐205, miR‐19a, miR‐19b, miR‐30b and miR‐20a) were shown to be valuable as diagnostic markers of squamous cell lung carcinoma (SQCLC) based on the drop in their circulating levels after surgical excision.^52^ Cazzoli et al also reported that exosomal miRNAs (miR‐200b‐5p, miR‐378a, miR‐139‐5p and miR‐379) derived from human plasma distinguished nodules from non‐nodules (97.5% sensitivity, 72.0% specificity) and exosomal miRNAs (miR‐629, miR‐30a‐3p, miR‐100, miR‐200b‐5p, miR‐154‐3p and miR‐151a‐5p) discriminated granulomas from LUAD (96.0% sensitivity, 60.0% specificity).^53^ Moreover, Zhou et al studied 265 subjects (141 LUAD patients and 124 healthy controls) and identified a set of exosomal miRNAs (miR‐19‐3p, miR‐21‐5p and miR‐221‐3p) for the discrimination of patients with LUAD from healthy subjects with sensitivity and specificity ranges of 67%‐73% and 66%‐80%, respectively.^54^ Additionally, Wu et al revealed that exosomal miR‐96 had the potential to be of diagnostic value in lung cancer patients.^22^ Another study reported that exosomal miR‐126 correlated with NSCLC and held substantial promise as a diagnostic biomarker for the disease.^55^ Exosomal miR‐23a and miR‐30b/30c have also been identified as possible biomarkers for the diagnosis of lung cancer.^26^, ^56^ Recently, a noteworthy study focused on early‐stage NSCLC and histologic heterogeneity, and found that exosomal miRNAs (miR‐let‐7b‐5p, miR‐let‐7e‐5p, miR‐23a‐3p and miR‐486‐5p) were promising diagnostic markers of stage I NSCLC patients with sensitivity and specificity values of 80.5% and 92.31%, respectively. It was also demonstrated that four LUAD‐specific miRNAs (miR‐181‐5p, miR‐30a‐3p, miR‐30e‐3p and miR‐361‐5p) and three SQCLC‐specific miRNAs (miR‐10b‐5p, miR‐15b‐5p and miR‐320b) were promising biomarkers with area under the curve (AUC) values of 0.936 and 0.911, respectively.^57^ In addition, exosomes are present in body fluids other than blood, and an increasing number of studies of NSCLC patients have focused on BAL and pleural fluid to identify diagnostic biomarkers. For example, only slight differences between miRNA expression in BAL‐ and plasma‐derived exosomes of lung cancer patients were identified in one study, and tumour‐specific miR‐122 was present in both.^20^ Moreover, exosomal miRNAs in pleural fluid (miR‐205‐5p, miR‐483‐5p, miR‐375, miR‐200c‐3p, miR‐429, miR‐200b‐3p, miR‐200a‐3p, miR‐203a‐3p and miR‐141‐3p) were demonstrated to favour the diagnosis of lung cancer over tuberculosis and other benign lesions.^58^, ^59^ Recently, Tamiya et al revealed that two exosomal miRNAs (miR‐182 and miR‐210) in pleural fluid may serve as promising biomarkers for the diagnosis of malignant pleural effusions from LUAD over benign pleural effusions with AUC values of 0.87 and 0.81, respectively.^60^ Exosomal miR‐200 in pleural effusions was also used to discriminate patients with LUAD from individuals with benign effusions.^61^ Interestingly, Lai et al^62^ used a mathematical model to establish a set of miRNAs (miR‐21, miR‐205 and miR‐155) for the early detection of NSCLC.

### 3.2 Exosomal miRNAs as predictive biomarkers in lung cancer

Acquired resistance to chemotherapy, radiotherapy and targeted therapies presents a major clinical challenge in the treatment of lung cancer.^63^ Exosomes can serve as vehicles for miRNAs that impact drug resistance and hence as biomarkers that predict therapeutic responses. To this end, exosomal miR‐221‐3p and miR‐222‐3p derived from plasma were associated with the response to osimertinib in EGFR‐mutated NSCLC.^64^ In addition, exosomal miR‐29a‐3p and miR‐150‐5p were identified as circulating biomarkers of delivered radiotherapy dose, which could possibly be exploited to predict response or toxicity.^65^ Exosomal miR‐208a in sera might influence the radiosensitivity of lung tumour cells by targeting p21.^21^ Moreover, Yuwen et al showed that serum exosomal miR‐146a‐5p might serve as a new biomarker predicting the efficacy of cisplatin in NSCLC and might be useful for real‐time monitoring of drug resistance.^41^ Exosomal serum miR‐222‐3p levels were recently proposed as a potential prognostic biomarker of gemcitabine sensitivity in NSCLC.^40^ Although clinical studies predicting therapy efficacy using exosomal miRNA are sparse, fundamental research studies are abundant. For example, exosomal miR‐4443 and miR‐100‐5p secreted by A549 cells responded to cisplatin treatment,^38^ and exosomal miR‐100‐5p was able to transfer cisplatin resistance to recipient cells by targeting rapamycin (mTOR) in vitro and in vivo.^39^ Moreover, exosomal miR‐96 derived from H1299 cells enhanced cisplatin resistance,^22^ while exosomal miR‐521 and miR‐373 were associated with cisplatin sensitivity in lung cancer cells.^16^ In summary, since exosomal miRNAs can provide information on donor cells and change the cellular state of target cells, they are positioned to regulate tumour resistance and could be used to monitor therapy response/relapse in personalized treatment.

### 3.3 Exosomal miRNAs as biomarkers of prognosis in lung cancer

Similar to other malignancies, tumour stage is the most important determinant of prognosis in NSCLC, along with other clinical and histologic variables.^66^ In recent years, exosomal miRNAs have also been proposed as prognostic factors in cancers, including lung cancer.^67^, ^68^ Dejima et al showed that the expression of exosomal miR‐21 and miR‐4257 in the plasma of NSCLC patients was significantly increased during recurrence.^69^ The predictive potential of these two miRNAs for recurrence was also validated in another large cohort, indicating that elevated plasma exosomal miR‐21 and miR‐4257 levels were linked with shorter disease‐free survival. Additionally, another study reported that the presence of serum exosomal miR‐146a‐5p correlated with higher recurrence rates in advanced NSCLC.^41^ Liu et al^70^ also demonstrated that plasma exosomal miR‐23b‐3p, miR‐10b‐5p and miR‐21‐5p were independent prognostic biomarkers of NSCLC. Furthermore, low levels of exosomal let‐7a‐5p were identified as a biomarker of poor survival in LUAD.^71^

### 3.4 Exosomal miRNA delivery system in lung cancer

Currently, a growing number of studies indicate that exosomes are ideal drug delivery vehicles. For therapy, drugs enclosed in exosomes are protected from degradation or damage due to their lipid bilayer membrane and nanoscale size.^72^ Moreover, exosomes are able to deliver their cargoes, including miRNAs, to specific recipient cells because of the ligands and peptides on their surface.^73^ In addition, exosomes are minimally immunogenic and toxic. A study by Srivastava et al^74^ reported an exosome delivery system with anti‐cancer activity against H1299 and A549 lung cancer cells. There are only a limited number of studies on exosomal miRNA delivery in lung cancer. Cortez et al^46^ reported a liposomal nanoparticle system by which they delivered miR‐34a mimics into a syngeneic LUAD mouse model and observed decreased numbers of macrophages, PD1‐expressing T cells and Tregs. Although these results are promising, we cannot ignore the problems of miRNA itself. One study reported that imported miRNA could lead to cellular toxicity.^14^ In addition, the mechanisms of miRNA packaging into exosomes are still unclear. Thus, applying exosomal miRNAs as delivery systems is challenging and needs further exploration.

## 4 CONCLUSIONS AND PERSPECTIVES

In conclusion, the discovery of exosomes and their multiple functions in cancer biology undoubtedly represents one of the most exciting findings in recent years. Based on current research, miRNAs can be selectively packaged into exosomes and play an important role in the proliferation, angiogenesis, metastasis and immunity of lung cancer. Moreover, exosomal miRNA profiling has the potential to be used as a diagnostic, predictive and prognostic tool for lung cancer and may provide a reliable and non‐invasive alternative to biopsies for monitoring recurrence and individual responses to therapies. However, some challenges should not be ignored. First, a guiding standard and updates on nomenclature, separation, characterization and functional analysis are provided by the ISEV, which should be implemented for the clinical application of exosomes [149, 150]. However, due to the heterogeneity in size and the probable subpopulations of exosomes,^75^ standard technologies must be established for the isolation and analysis of exosomes and their miRNA content. Second, our current knowledge of exosome biogenesis, sorting mechanisms for miRNAs into exosomes, secretion and uptake mechanisms of exosomal miRNA is still too limited to allow for clear conclusions to be drawn on how exosomal miRNAs interact with recipient cells. The specific molecules and mechanisms of the exosomal miRNA delivery system require further exploration. Third, although exosomal miRNAs have been demonstrated to be transferred to recipient cells, the fate of exosomes and exosomal miRNAs remains incompletely understood. Therefore, superresolution and tracking techniques as well as new in vivo models to follow exosomes should be developed. Fourth, large‐scale studies with patient stratification are urgently needed to achieve reproducible results and confirm the clinical safety and efficacy of exosomal miRNAs as therapeutic agents.

## CONFLICTS OF INTEREST

None.

## AUTHOR CONTRIBUTIONS

CH and JC wrote the manuscript; SM and GT reviewed and edited the manuscript before submission; and CL prepared the figures.

## Data Availability

The data that support the findings of this study are available from the corresponding author upon reasonable request.
